# Development of a Seismic Detection Technology for High-Speed Trains Using Signal Analysis Techniques

**DOI:** 10.3390/s20133708

**Published:** 2020-07-02

**Authors:** Jae Sang Moon, Mintaek Yoo

**Affiliations:** 1 Structural Department, Yooshin Engineering Corporation, Seoul 06252, Korea; mjaesang@gmail.com; 2 Railroad Structure Research Team, Korea Railroad Research Institute (KRRI), Uiwang 16105, Korea

**Keywords:** earthquake awareness, seismic detection technology, high-speed train, signal analysis

## Abstract

As the occurrence of earthquakes is increasing in South Korea, the earthquake early warning (EEW) system becomes indispensable for the protection of high-speed railways. Although the importance of EEW system has been increasing, the number of installed seismic accelerometers in South Korea is not sufficient to provide rapid information. This study uses a stochastic signal analysis technique to utilize the smartphone sensors for the rapid EEW system. From the train vibration data from the low fidelity on-board accelerometer, the virtual earthquake detection data in the train by smartphone sensor has been constructed. To analyze the stochastic characteristics of the constructed data, the short time Fourier transform (STFT) approach has been applied. The study’s overall objective is to offer stochastic approaches that provide effective analysis of the low fidelity sensor data, such as smartphone sensor data, for the rapid EEW system.

## 1. Introduction

In South Korea, earthquake awareness is increasing due to a series of consecutive magnitude 5.0 earthquakes, including the 2016 Gyeongju [1] and 2017 Pohang [2] earthquakes. In particular, for railway facilities—which are a component of the large-scale, national infrastructure—an earthquake causes the bending of railways, roadbed settlement, and landslides, which may result in train derailments or collision and in turn human casualties and substantial property damage. Therefore, an early warning system is required to rapidly slow down or stop the operation of trains in the event of an earthquake. For the high-speed Korea Train Express (KTX) railway in South Korea, seismic detection systems have been constructed and operated by seismic accelerometers that are installed at 20–30 km intervals in major piers and tunnels, as well as by borehole accelerometers in the free fields separated from tracks and pier structures [3]. The current warning systems for domestic high-speed railways, however, have slow response times because the operation of trains is only determined after train operators are notified of emergencies through wired communication. In addition, the occurrence of an earthquake cannot be detected if a train passes through an area without a seismometer. As seismometers are costly and difficult to maintain, it is difficult to install and operate many of them, and thus dead-zones in the network arise. 

To address this challenge, various studies have been conducted on seismometer networks overseas. QuakeCatcherNetwork (QCN: http://quakecatcher.net) constructed a global seismic observation network using micro-electromechanical system (MEMS) accelerometers attached to desktops, laptops, and USB devices that are fixed to the floor [4,5,6,7]. QCN is now operating the network in the Southern California Earthquake Center (SCEC) and the Incorporated Research Institutions for Seismology (IRIS). Community Seismic Network (CSN: http://csn.caltech.edu) is constructing and operating a community seismic observation network by providing participants with free USB seismometers, which were developed by the California Institute of Technology in the United States and can be connected to computers [8,9,10,11,12,13,14]. The most recent study is MyShake (http://myshake.berkeley.edu), which developed a seismic observation network application using smartphone sensors and has more than 300,000 registered users worldwide now. This application can alert users to an earthquake in advance due to its early warning functions. MyShake has been evaluated to be superior to ShakeAlert (https://www.shakealert.org)—an earthquake early warning system in the western United States—in terms of both speed and accuracy [15,16,17]. Additionally, the Pacific Gas and Electric Company (PG&E) is pursuing a project to build a dense seismic observation network by attaching accelerometers to more than 300,000 gas and electricity meters in the Bay Area of the western United States [18]. The investigation of earthquake early warning systems has been an international effort. In 1992, the Urgent Earthquake Detection and Alarm System (UrEDAS) was developed in Japan and has since been used in the operation of the Shinkansen [19,20]. UrEDAS can issue an early warning by detecting P-waves approaching railways at nearby seismic stations. For the JR Earthquake Early Warning (EEW) system, operated by the East Japan Railway Company, the seismic stations are installed along the coast and the railways.

This study applied a rapid EEW algorithm using on-board accelerometers in trains as alternatives to seismometers to inform the earthquake response of train operation. On-board accelerometers, however, measure not only earthquakes but also the vibration loads of trains. Thus, a strategy to distinguish normal vibration load from seismic vibrations is necessary. To this end, the vibration and earthquake data measured during train operation were analyzed, and the seismic detection of on-board accelerometers was evaluated.

In brief, the study was performed as follows. First, vibration measurement data for a train in the event of a virtual earthquake were constructed by superimposing train vibration measurements and domestic earthquake data. The vibration measurement data were obtained from the vibration measurement sensor installed on a train and an Android smartphone during Korea Train Express (KTX) operation in a particular rail segment. Pohang, Gyeongju, and Hachinohe earthquake data were used as the simulated earthquake data. The constructed data were subjected to short time Fourier transform (STFT) and compared with the train vibration data. Based on this comparison, we investigated the practicality and effectiveness of detecting an earthquake on a train by this method, particularly with regards to the value of smartphone sensors for this application.

## 2. Materials and Methods

### 2.1. Train Data Collection

**Figure 1:**
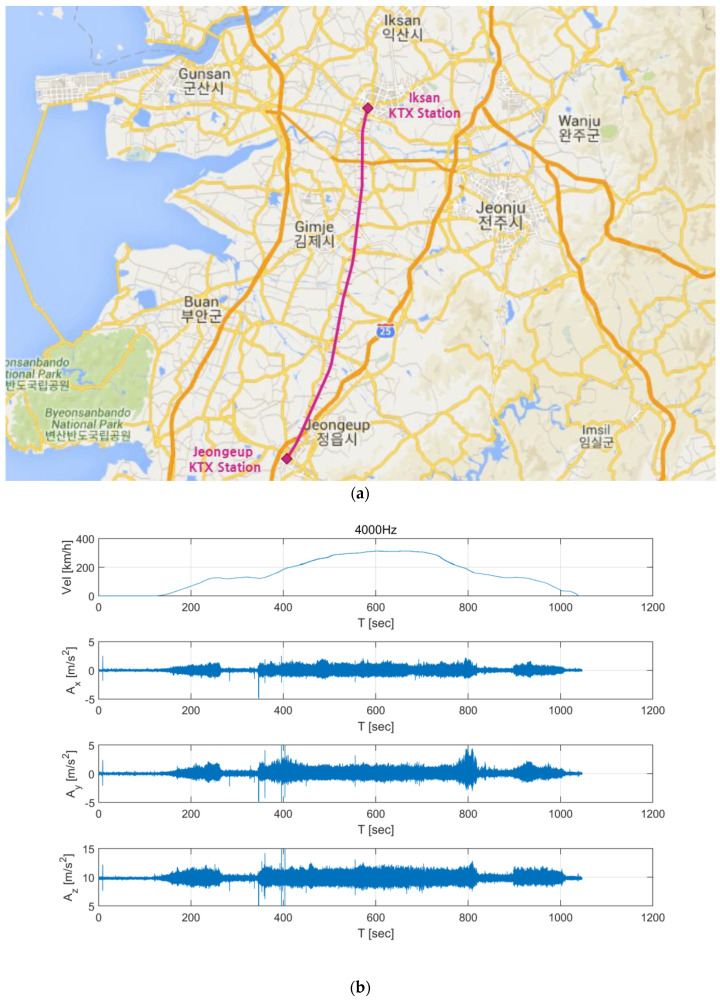

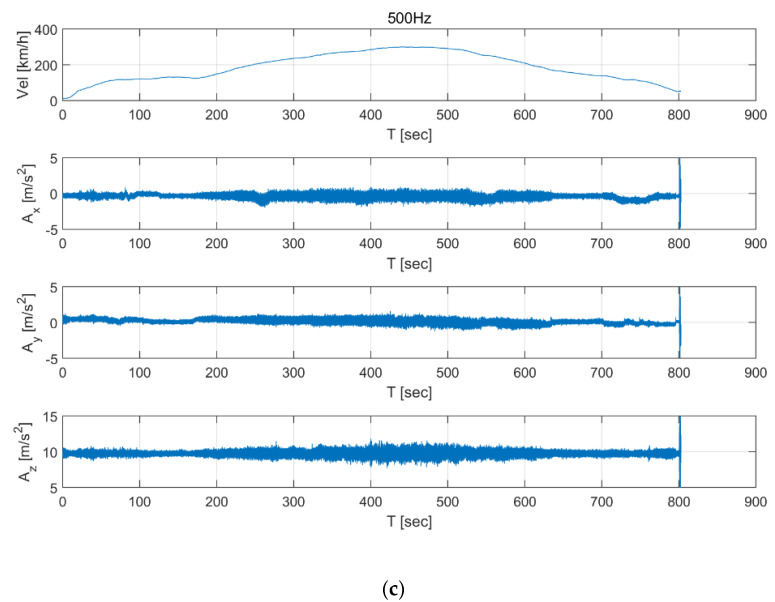
Korea Train Express KTX Data. (**a**) Travel route of the train for which train vibration data was collected. (**b**) Acceleration data collected from the installed vibration sensor. (**c**) Acceleration data collected from an Android smartphone located in the train car.

The train data were measured while a KTX train traveled from Iksan to Jeongeup; the route is shown in Figure 1a. Acceleration values in the longitudinal (*x*), transverse (*y*), and vertical (*z*) directions were measured using a vibration sensor installed in a train car and a smartphone sensor located in the car. The sampling frequencies of the sensors were 4000 and 500 Hz, respectively. Figure 1b shows the acceleration time series measured by the installed sensor, and Figure 1c shows the acceleration time series measured by the smartphone. In this paper, these acceleration time series are expressed as $A_{i}^{4000\mathrm{Hz}}\left(t\right)\left(i=x,y,z\right)$ and $A_{i}^{500\mathrm{Hz}}\left(t\right)\left(i=x,y,z\right)$, respectively.

### 2.2. Seismic Data Collection

**Figure 2:**
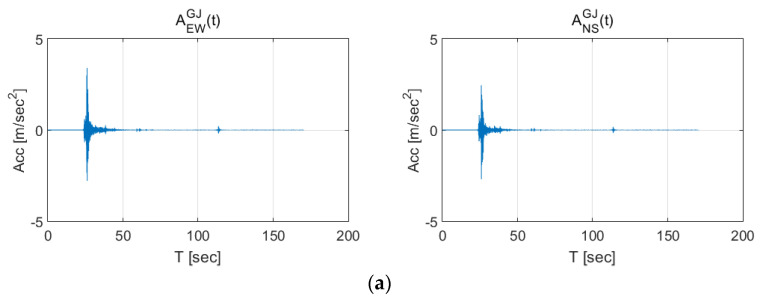

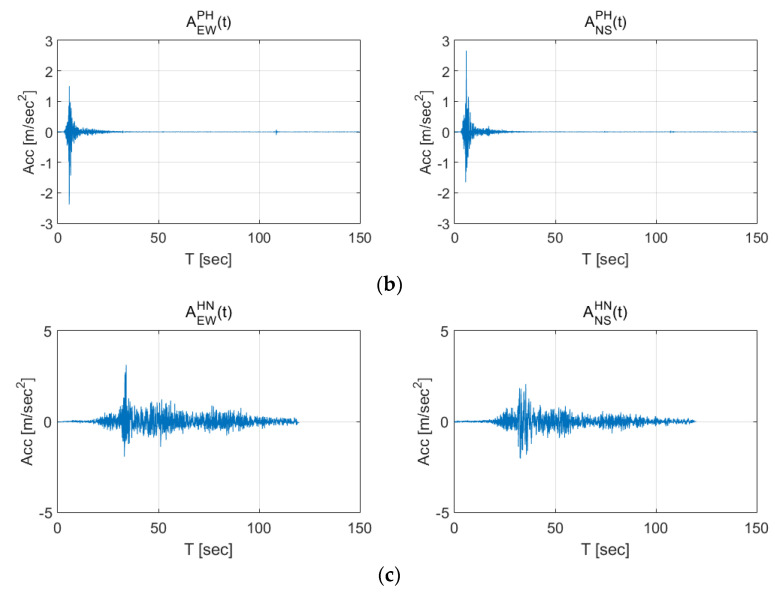
Seismic activities in (**a**) Gyeongju (2016), (**b**) Pohang (2017), and (**c**) Hachinohe (1994).

For the earthquake data, the acceleration histories over time measured during the Gyeongju, Pohang, and Hachinohe earthquakes were used. The 2016 Gyeongju earthquake had a magnitude of 5.8 on the Richter scale, the 2017 Pohang earthquake had a magnitude of 5.4, and the 1994 Hachinohe earthquake had a magnitude of 7.7. Figure 2 shows the accelerations measured in each city when the earthquakes occurred. These measurements were performed by seismic stations in fixed orientations (east-west and north-south directions). Thus, they are expressed as $A_{i}^{\mathrm{GJ}}\left(t\right)\left(i=\mathrm{EW},\mathrm{NS}\right)$, $A_{i}^{\mathrm{PH}}\left(t\right)\left(i=\mathrm{EW},\mathrm{NS}\right)$, and $A_{i}^{\mathrm{HN}}\left(t\right)\left(i=\mathrm{EW},\mathrm{NS}\right)$, respectively, in this paper. As shown in Figure 2, the Gyeongju and Pohang earthquakes exhibit rapidly decreasing acceleration after the initial maximum acceleration. However, the Hachinohe earthquake exhibited an acceleration of approximately 1 m/s^2^ for 100 s before and after the maximum acceleration.

### 2.3. Superimposing Seismic Data on Train Data

**Figure 3:**
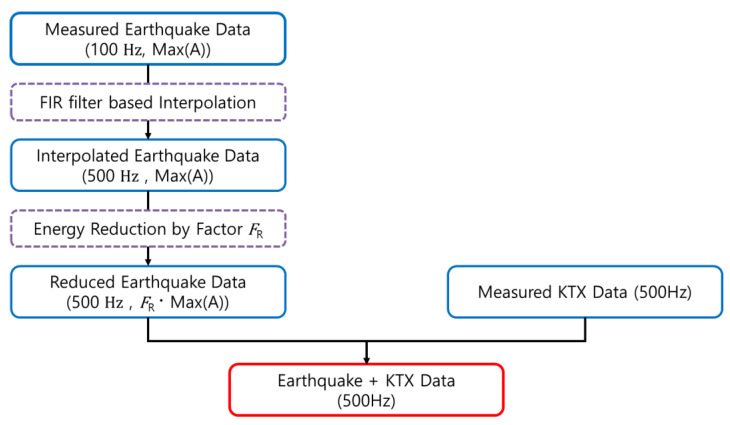
Data synthesis flow chart.

The simplest method to generate the simulated data from a train in the event of an earthquake is to superimpose the train measurement data and the earthquake measurement data. As described above, however, overlaying the two datasets is difficult because the sampling rates of the train data and the earthquake data differ. Moreover, if the measured earthquake occurs on a train track, the train is likely to derail. Therefore, it is unrealistic to assume that simply superimposing the seismic and train data will be sufficient to simulate earthquake measurement data from a train. To address these problems, two adjustment steps have performed as follows (Figure 3).

First, the earthquake data was up-sampled according to the sampling rate of the train data. Appropriate up-sampling must reflect the characteristics of the original data. If the measurement data are directly up-sampled, however, the high-frequency domain elements of the data may not be accurately estimated. Therefore, in this study, the sampling rate of the earthquake data was first changed from 100 to 500 Hz by applying an interpolation algorithm based on a finite impulse response filter to the original data.

After adjusting the sampling rate of the earthquake data, the energy of the earthquake was reduced. The seismic accelerations were selected as from 1 and 2 m/s^2^ according to the 500 years and 2400 years return period of earthquake in Korea seismic design code [21].

### 2.4. Data Analysis—Short Time Fourier Transform

The STFT is a commonly used method for the identification of changes in the characteristics of time-history data over time [22,23,24,25,26]. The typical power spectral density (PSD) function of time series *A*(*t*) with duration *T* is

(1) $$PSD_{A}\left(f\right)={\left|\hat{A}\left(f\right)\right|}^{2},$$

where

(2) $$\hat{A}\left(f\right)=FFT\left(A\left(t\right)\right),$$

and where *t* is time such that 0≤t≤T and $\hat{A}\left(f\right)$ is the Fourier transform of time series A(t). The above equation is valid when A(t) is a stationary process in a given time interval 0≤t≤T. If the characteristics of the time series change over time, the above equation can reveal the averaged frequency domain characteristics of the entire dataset. However, it is difficult to express the trend of frequency domain characteristics at different time. Thus, STFT was used in this study to analyze the characteristics of time series as time changes. STFT is defined as follows:

(3) $$PSD_{A}\left(f\right)=\{\begin{array}{cc} PSD_{A,1}\left(f\right) & 0\leq t\leq T_{1}\\ \begin{array}{c} PSD_{A,2}\left(f\right)\\ \vdots \end{array} & \begin{array}{c} T_{1}\leq t\leq T_{2}\\ \vdots \end{array}\\ PSD_{A,N_{w}}\left(f\right) & T_{N-1}\leq t\leq T_{N_{w}} \end{array},$$

where

(4) $$PSD_{A,j}\left(f\right)={\left|{\hat{A}}_{i}\left(f\right)\right|}^{2},$$

**Figure 4:**
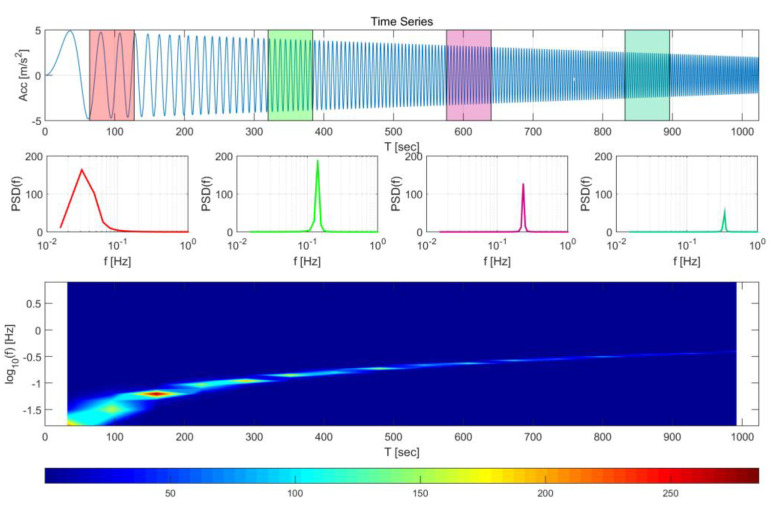
Example of a short-time Fourier transform (STFT) and associated spectrogram.

$PSD_{A,i}\left(f\right)$ is the PSD for a specific time interval $T_{j-1}\leq t\leq T_{j}$ and the entire PSD function is expressed as a function that varies with time interval. Figure 4 shows the results of STFT on a signal whose amplitude and frequency change over time. Notably, unlike the typical PSD, the characteristics that change over time can be observed by this method. The graph which represents the PSD change over time based on the STFT is referred to as a spectrogram.

## 3. Results

### 3.1. Scaled Seismic Data

**Figure 5:**
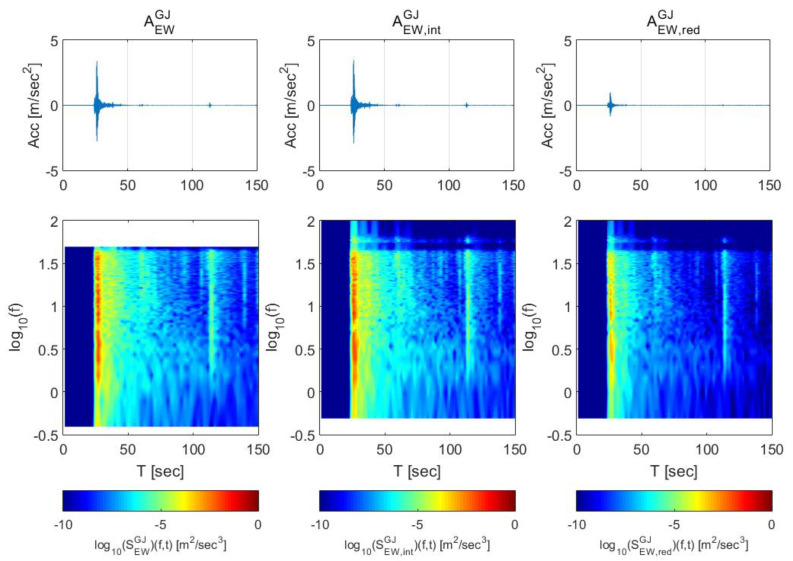
Gyeongju earthquake data adjustment—original data (100 Hz), interpolated data (500 Hz), and scaled data (max 1 m/s^2^), from the left.

Figure 5 shows the STFT of original data (left), interpolated data (center), and scaled data (right). The STFT of the interpolated data shows the high-frequency domain characteristics that had not been observed in the original data while other characteristics of the original data were maintained. After the reduction process, the maximum energy level has been lower than that of the original data.

**Figure 6:**
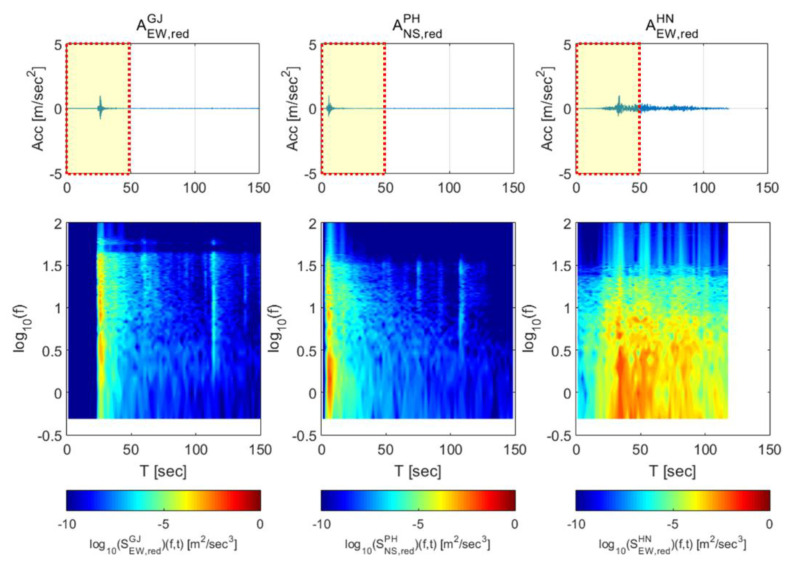

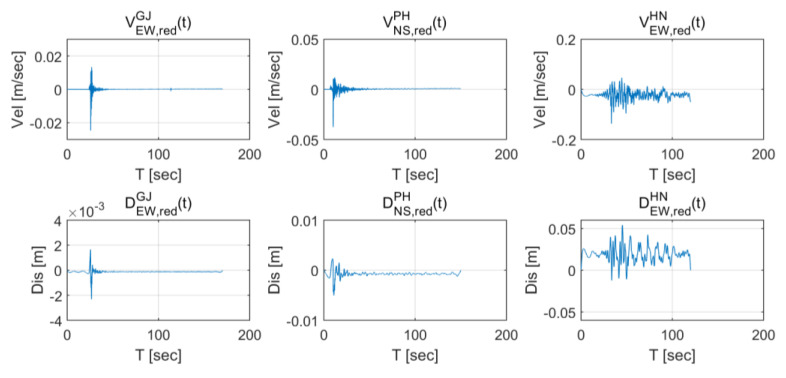
Adjusted earthquake data used for the study. The Gyeongju and Hachinohe earthquakes (**far left** and **far right**, respectively) are in the east-west direction. The Pohang (**center**) earthquake is in the north-south direction.

**Figure 7:**
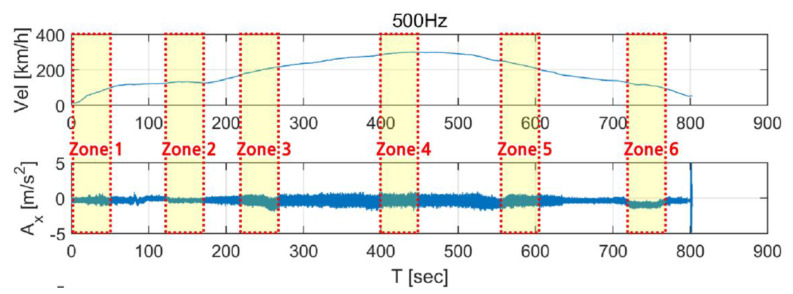
Zones selected from earthquake data for superimposed with train data.

Figure 6 shows section of the earthquake data used to synthesize train-measured seismic data. High-frequency domains were generated in all data compared to the original data, and the overall energy level was reduced. These adjusted earthquake data were superimposed with the train data. The velocity and displacement time history were obtained by integrating acceleration time history. As shown in Figure 7, the sections of the first acceleration (0–50 s, zone 1), constant speed (130–180 s, zone 2), second acceleration (220–270 s, zone 3), maximum speed (400–450 s, zone 4), first deceleration (550-600 s, zone 5), and second deceleration (720–770 s, zone 6) were selected to “occur” during train operation. The train data were then superimposed with the data of each earthquake for the first 50 seconds (marked sections in Figure 6). As train derailment is associated with the vibration load applied in the transverse direction, the transverse direction train data were superimposed with the earthquake data for subsequent investigations.

### 3.2. Spectral Characteristics of Train Data

**Figure 8:**
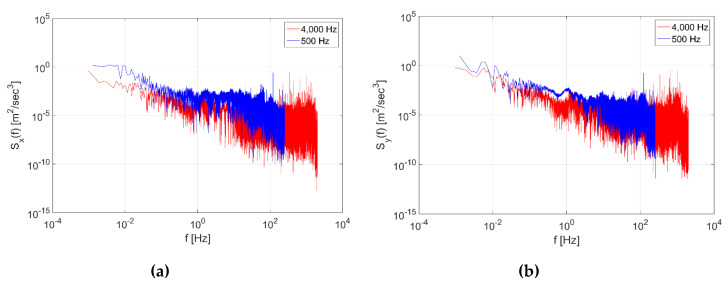
Power Spectral Density (PSD) of Korea Train Express (KTX) Data in the (**a**) longitudinal direction and (**b**) transverse direction.

The train data measured by the Android smartphone were compared to those measured by the sensor installed in an actual train to examine their validity and to confirm whether they reflected the actual train characteristics. Figure 8 shows the PSD of the KTX data. In both the longitudinal and transverse directions, below 10 Hz, the PSD of the 500 Hz data was skewed towards higher frequencies by 10 Hz compared to the 4000 Hz data. For the 4000 Hz data, however, their PSD values could be reduced because they included the data measured in sections where the train was stopped.

**Figure 9:**
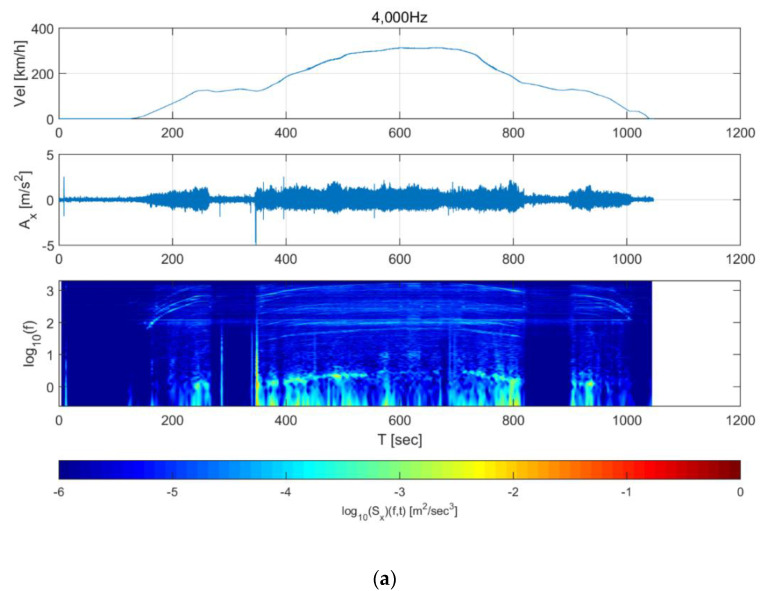

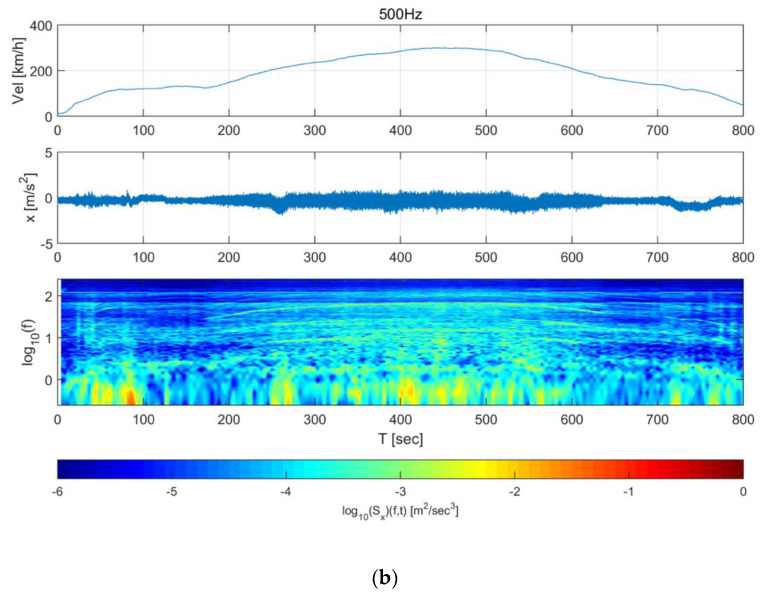
Short-time Fourier transform of Korea Train Express (KTX) data in the longitudinal direction for (**a**) 4000 Hz data and (**b**) 500 Hz data.

**Figure 10:**
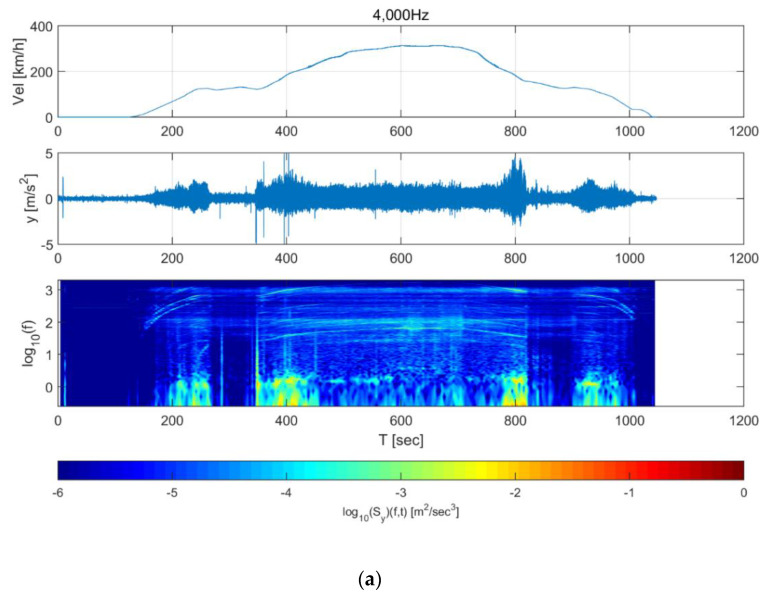

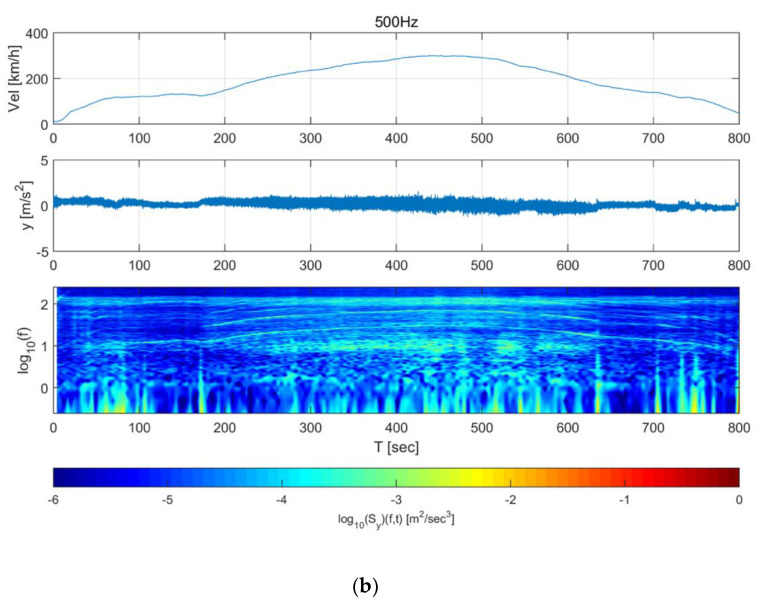
Short-time Fourier transform of Korea Train Express (KTX) data in the transverse direction for (**a**) 4000 Hz and (**b**) 500 Hz data.

Figure 9 and Figure 10 show the spectrograms of the train data. For the 40,000 Hz longitudinal direction data (Figure 9a), energy rose in all frequency domains from 120 s, and high energy was observed in the 100 Hz domain throughout. This 100 Hz energy is likely associated with equipment that supplies the power for acceleration. In addition, it was observed that the frequency of some energy increased or decreased with the increase or decrease in speed. This may be due to the vibration of the wheels and gears, which are directly related to the speed of the train. Finally, the energy decreased in all frequency domains when the train was traveling at a constant speed. For the 500 Hz data, the energy in the 100 Hz domain—which was also observed in the 4000 Hz data—and the change in frequency due to the train speed can be observed in Figure 9b. In the 500 Hz data, however, the energy reduction when the train was travelling at a constant speed was not significant compared to the 4000 Hz data. The transverse direction data in Figure 10 show similar trends to the longitudinal direction data in Figure 9. The measurement characteristics of the train sensor and the smartphone sensor can also be examined from Figure 9 and Figure 10. 

### 3.3. Spectral Characteristics of Seismic Data

**Figure 11:**
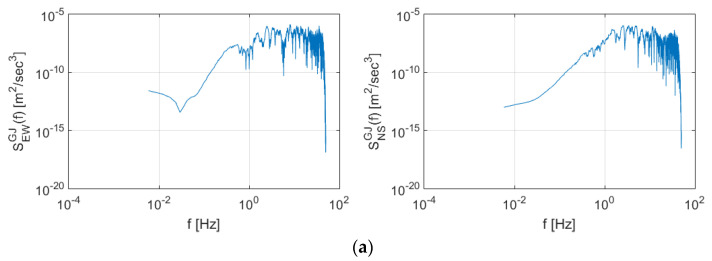

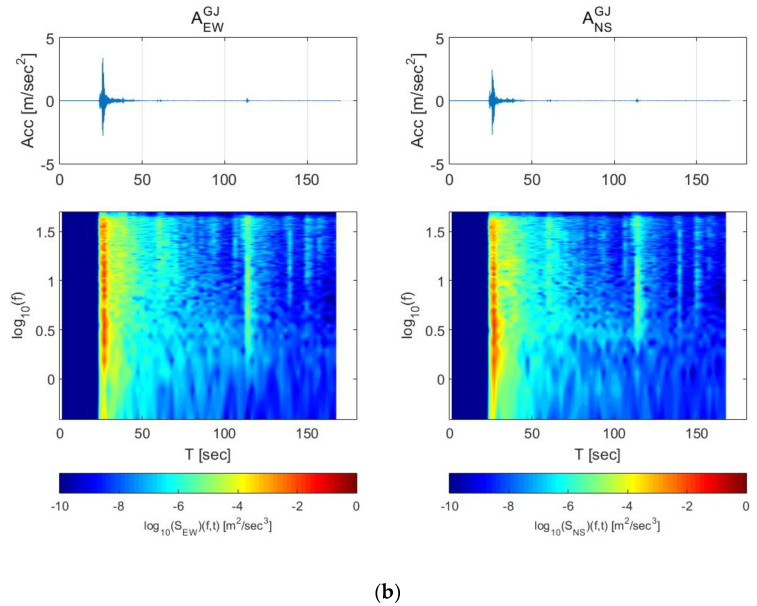
Spectral characteristics of the Gyeongju earthquake presented as (**a**) Power Spectral Density (PSD) and (**b**) spectrogram.

Figure 11 shows the Power Spectral Density (PSD) and spectrogram for the Gyeongju earthquake data. High energy was observed in the section above 1 Hz in both the east-west and north-south directions. After the period of maximum energy (>30 s), surges in energy generation were observed at 60 and 110 s in frequency domains greater than 3 Hz. 

**Figure 12:**
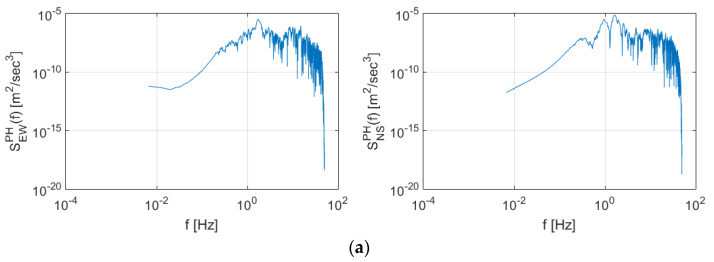

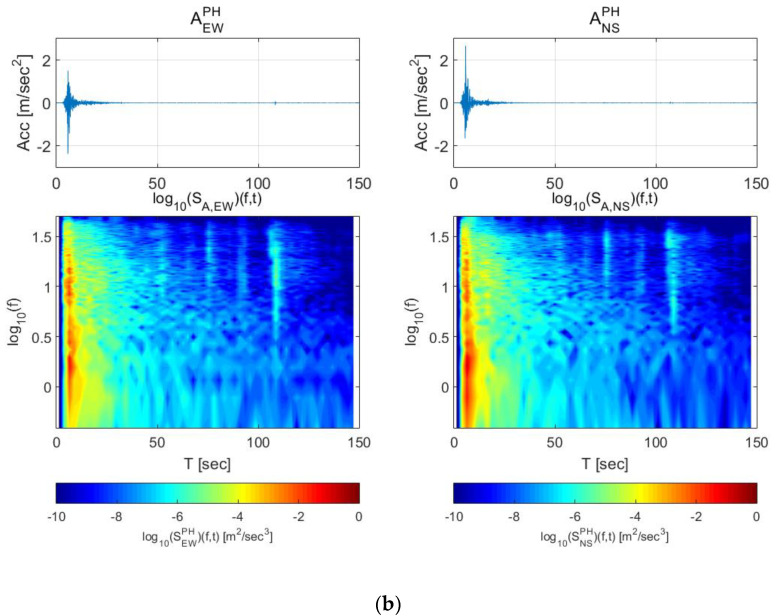
Spectral characteristics of the Pohang earthquake presented as (**a**) Power Spectral Density (PSD) and (**b**) spectrogram.

Figure 12 shows the PSD and spectrogram of the Pohang earthquake data. High energy was also observed for frequency domains less than 1 Hz in both the east-west and north-south directions. In addition, the duration of the maximum energy production was longer than that of Gyeongju earthquake. The additional energy generated, however, was observed in domains greater than 3 Hz, similar to Gyeongju earthquake. Furthermore, compared to the Gyeongju earthquake, the frequency domain exhibiting high energy was relatively low.

**Figure 13:**
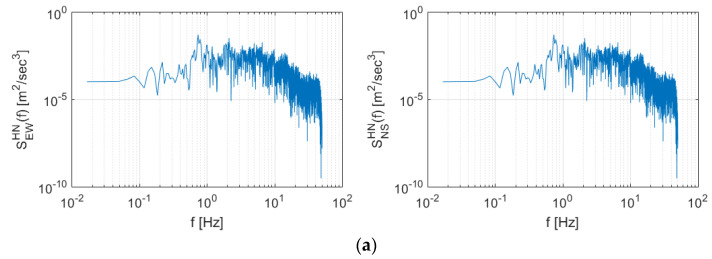

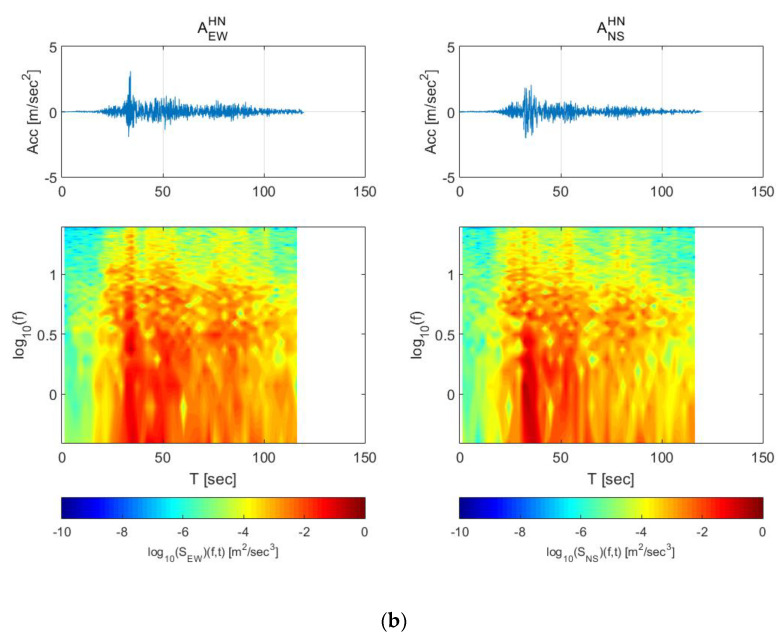
Spectral characteristics of the Hachinohe earthquake presented as (**a**) Power Spectral Density (PSD) and (**b**) spectrogram.

Figure 13 shows the PSD and spectrogram of the Hachinohe earthquake data. Compared to other seismic datasets, High energy was also observed in the frequency domain less than 1 Hz in both the east-west and north-south directions. In addition, the duration of maximum energy production was longer than that for the Gyeongju earthquake. The additionally generated energy, however, was observed in domains greater than 3 Hz, similar to the Gyeongju and Pohang earthquakes. Compared with the Gyeongju and Pohang earthquake, the frequency domain with high energy was relatively low.

### 3.4. Spectral Characteristics of Train-Measured Seismic Data

**Figure 14:**
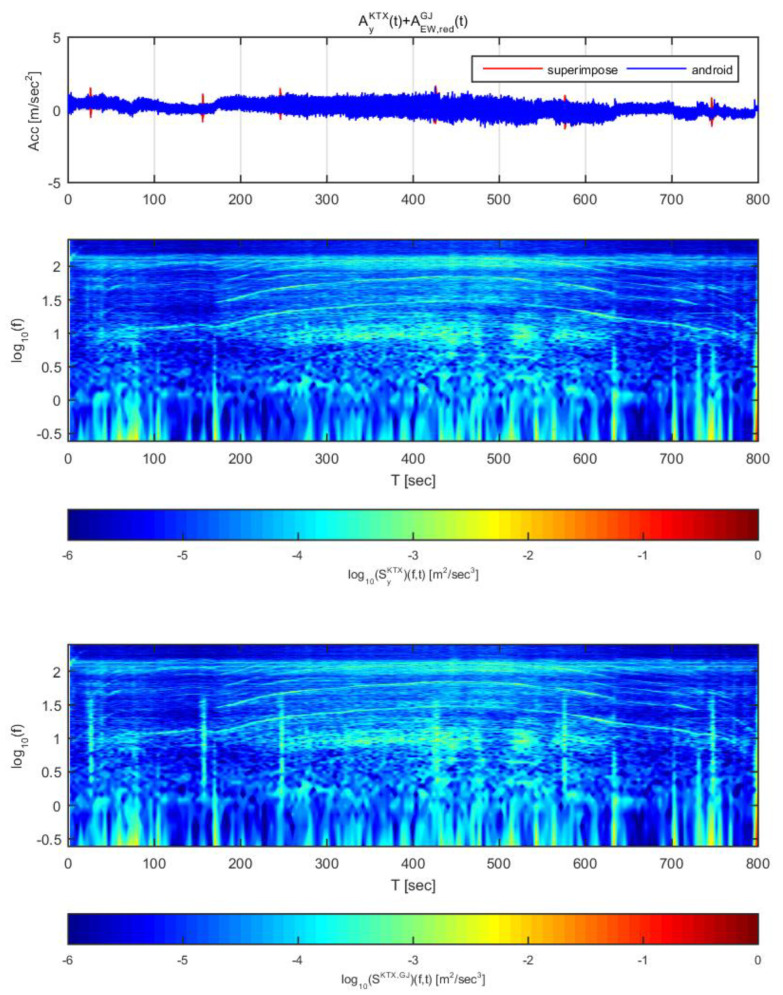
Combination of Korea Train Express (KTX) transverse direction data and Gyeongju earthquake east-west direction data (adjusted to 1 m/s^2^).

**Figure 15:**
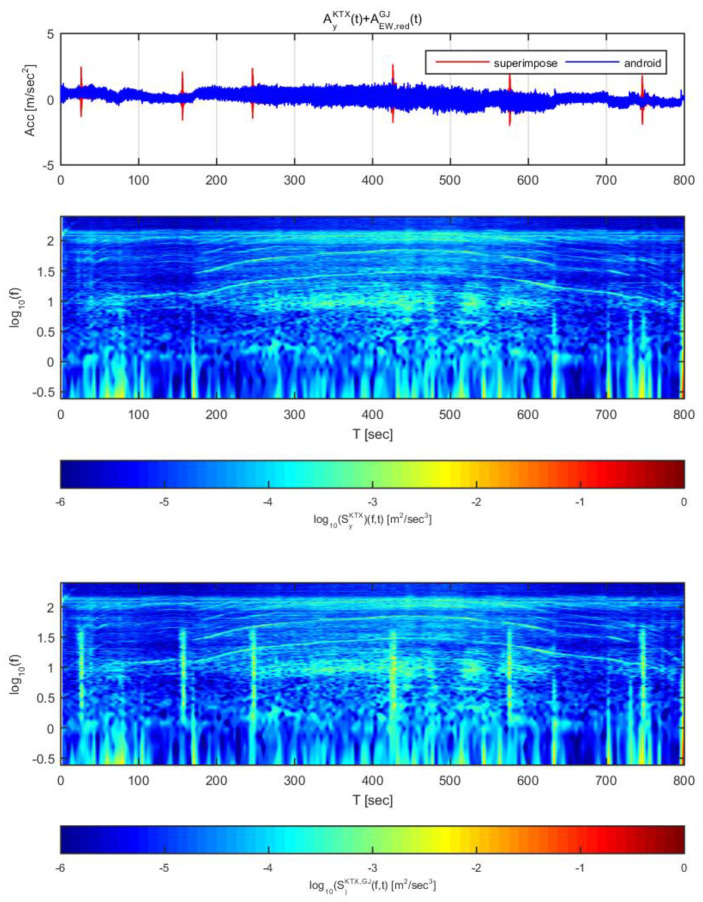
Combination of Korea Train Express (KTX) transverse direction data and Gyeongju earthquake east-west direction data (adjusted to 2 m/s^2^).

Figure 14 and Figure 15 show the time histories and spectrograms of the transverse direction train data and the adjusted Gyeongju earthquake data. Total length of time history is 800 s and the size of window is 4.096 s, Figure 14 displays the results for the earthquake data in the east-west direction adjusted to 1 m/s^2^, whereas Figure 15 shows those adjusted to 2 m/s^2^. In Figure 14, only the constant speed section exhibited differences in the time history; no other significant differences were observed. With regards to the spectrogram, only the constant speed section exhibited a significant difference, similar to the time history. This is likely because the frequency domain of the energy that affects the transverse direction acceleration in the devices used to measure the acceleration and gear system of the in the high-speed section was very similar to that of Gyeongju earthquake, and the acceleration energy level of the train data was higher. When the overall magnitudes of displacement and energy were considered, it was determined that an earthquake with spectral characteristics similar to those of Gyeongju earthquake is relatively difficult to detect by this approach when the acceleration is 1 m/s^2^. However, at this energy level, train derailment is extremely unlikely. 

In Figure 15, all train acceleration zones exhibited significant differences in time history. Significant differences were also observed in the spectrogram compared to Figure 14. For the first acceleration and second deceleration sections, however, the train data showed a trend similar to that of the superimposed data. This is likely because the energy generated by the gear and braking system used in the low speed section was similar to the energy of the Gyeongju earthquake in terms of magnitude and duration. Based on this result, the earthquake can be detected at an acceleration of 2 m/s^2^ with displacement and spectrograms, though there is no significant derailment risk because the earthquake energy is low.

**Figure 16:**
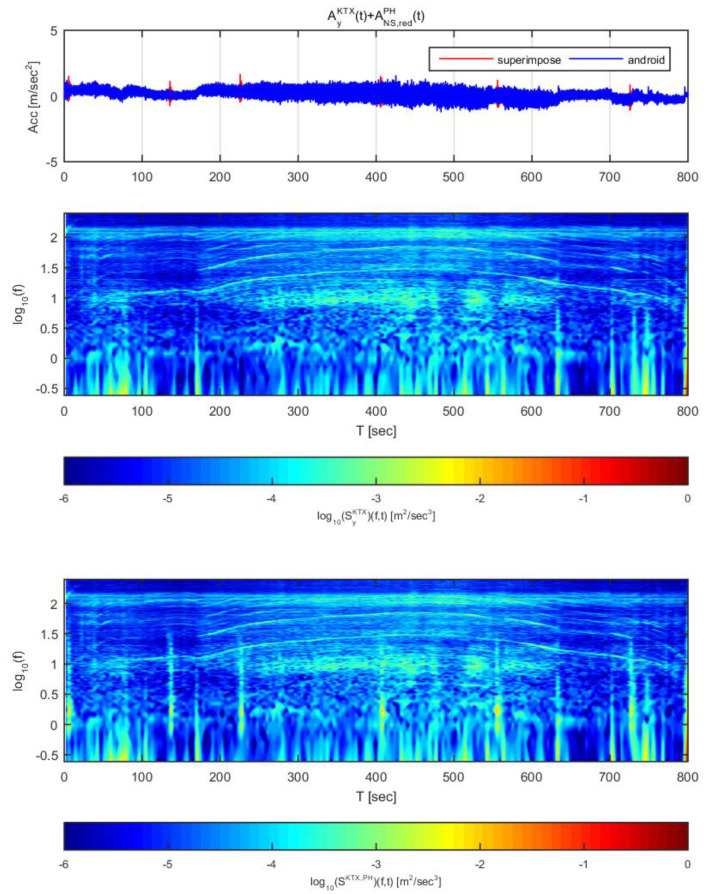
Combination of Korea Train Express (KTX) x-direction data and Pohang earthquake north-south direction data (adjusted to 1 m/s^2^).

**Figure 17:**
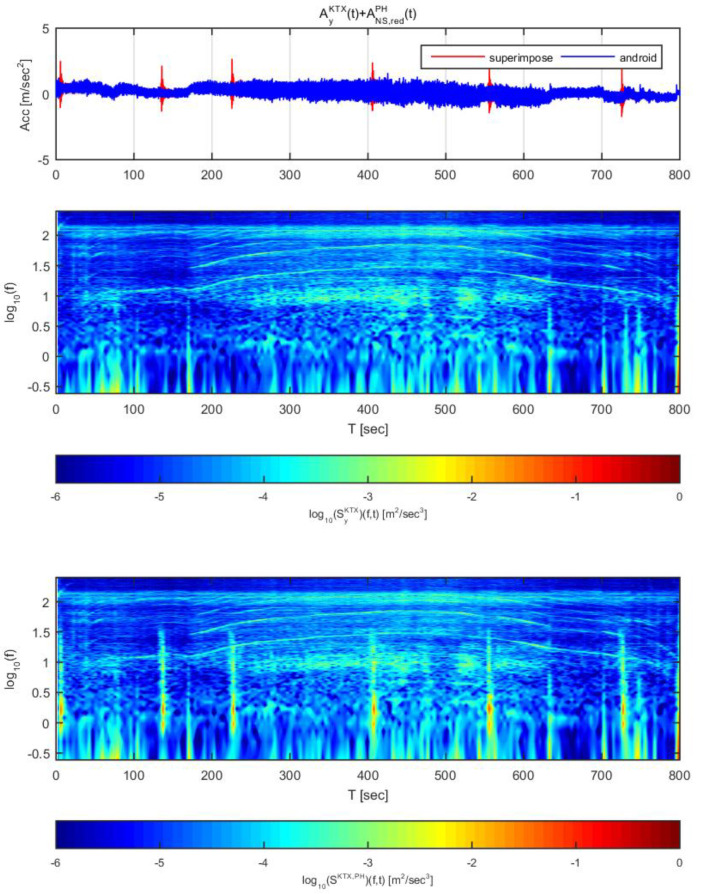
Combination of Korea Train Express (KTX) x-direction data and Pohang earthquake north-south direction data (adjusted to 1 m/s^2^).

Figure 16 and Figure 17 show the time histories and spectrograms of the longitudinal direction train data superimposed with the adjusted Pohang earthquake data. Figure 16 used the earthquake data in the north-south direction adjusted to 1 m/s^2^, whereas Figure 17 used the earthquake data adjusted to 2 m/s^2^. In Figure 16, only the constant speed section exhibited a significant difference in time history; the other sections showed no significant differences in acceleration. With regards to the spectrogram, only the constant speed and second acceleration sections exhibited significant differences. This result is similar to the results obtained for the Gyeongju earthquake data. 

In Figure 17, all sections exhibited significant differences in acceleration history over time. Significant differences were also observed in the spectrogram, which differs from when the acceleration was 1 m/s^2^. In particular, in the domain of 1–3 Hz, the energy level was notably amplified. The Pohang earthquake may be difficult to detect by this approach at an acceleration of 1 m/s^2^ because the energy level is not as high as the Gyeongju earthquake, though there is very low risk of derailment at this level. When the Pohang earthquake is scaled to an acceleration of 2 m/s^2^, it can be detected with displacement curves and spectrograms.

**Figure 18:**
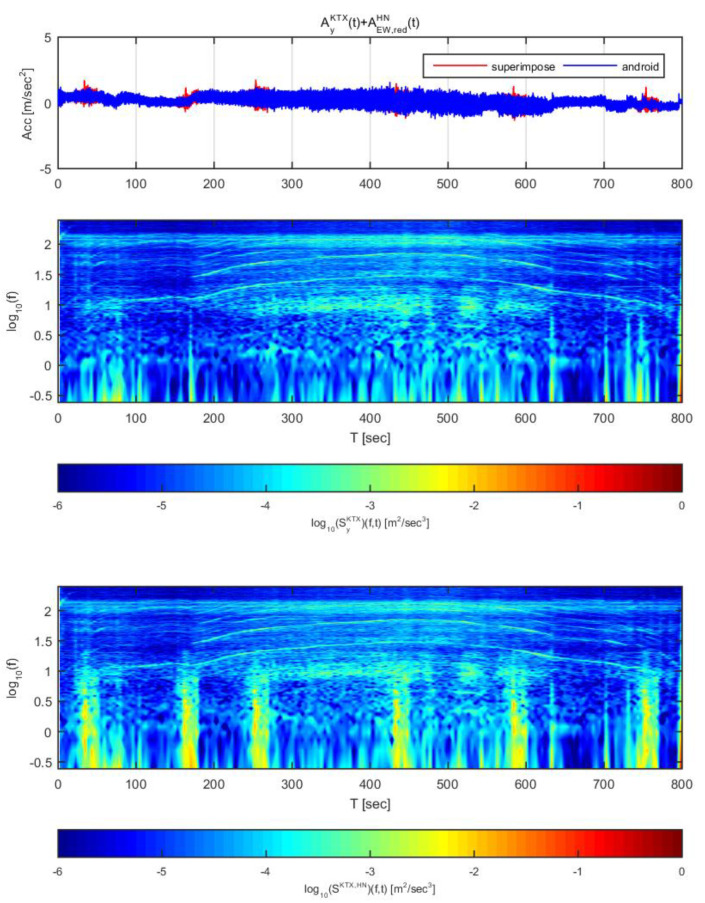
Combination of Korea Train Express (KTX) transverse direction data and Hachinohe east-west direction data (adjusted to 1 m/s^2^).

**Figure 19:**
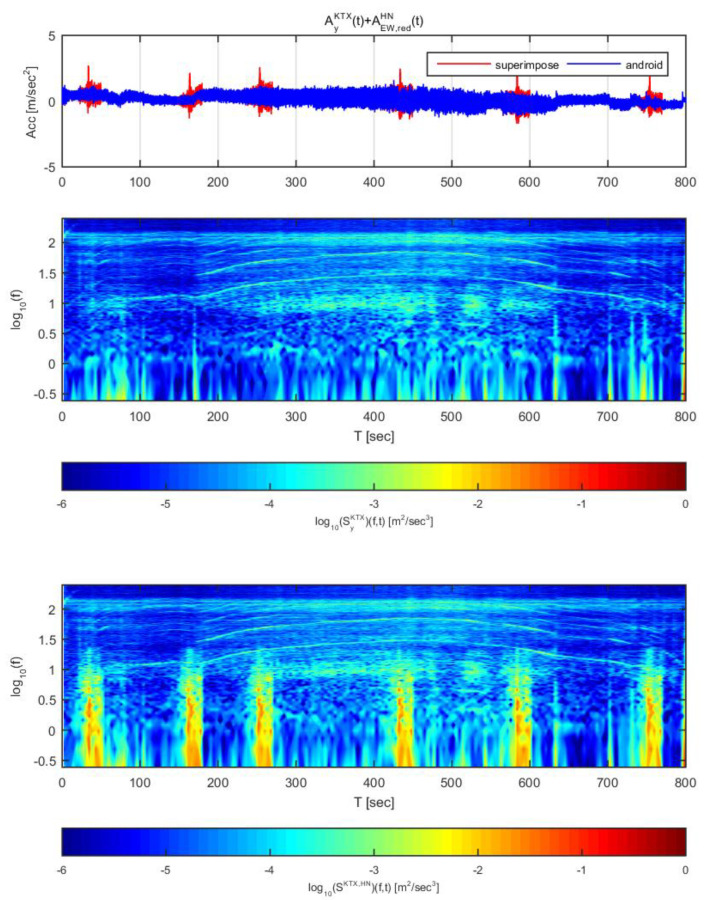
Combination of Korea Train Express (KTX) transverse direction data and Hachinohe east-west direction data (adjusted to 2 m/s^2^).

Figure 18 and Figure 19 show the time histories and spectrograms of the transverse direction train data and the adjusted Hachinohe earthquake data. Figure 18 used the earthquake data in the east-west direction adjusted to 1 m/s^2^, and Figure 19 used the data adjusted to 2 m/s^2^. In Figure 18, only the constant speed section exhibited a difference in time history; the other sections showed no significant differences, similar to the other earthquakes. With regards to the spectrogram, a clear difference was detected, unlike for the two earthquakes described above. In particular, the seismic energy that was significantly greater than that of the train and was observed in the low frequency range below 1 Hz, where energy increases due to the mechanical elements of the train. This detection is possible due to the high energy and long duration of the Hachinohe earthquake. 

In Figure 19, all sections exhibited significant differences in both acceleration time history and spectrogram, similar to the results obtained for the other earthquakes. Taken together with the results shown in Figure 18, it was confirmed that an earthquake similar to the Hachinohe earthquake can be detected by this approach even if its amplitude is small. In particular, the earthquake can be detected due to the high energy of the earthquake despite the distribution of the mechanical energy of the train in the low-frequency section.

### 3.5. Comparison with Continuous Wavelet Transform

To discuss the suggested methodology, the data has been analyzed using the wavelet transform (WT) and compared the analysis results with the STFT results. The wavelet transform has been widely used in the area of seismic analysis [27,28,29]. Since the method uses various functions instead of sine and cosine functions, it has advantage on modeling abrupt signals or nonlinear datasets [30,31,32]. In this study, the continuous wavelet transform using Morlet mother wavelet has been applied to the combined datasets. 

**Figure 20:**
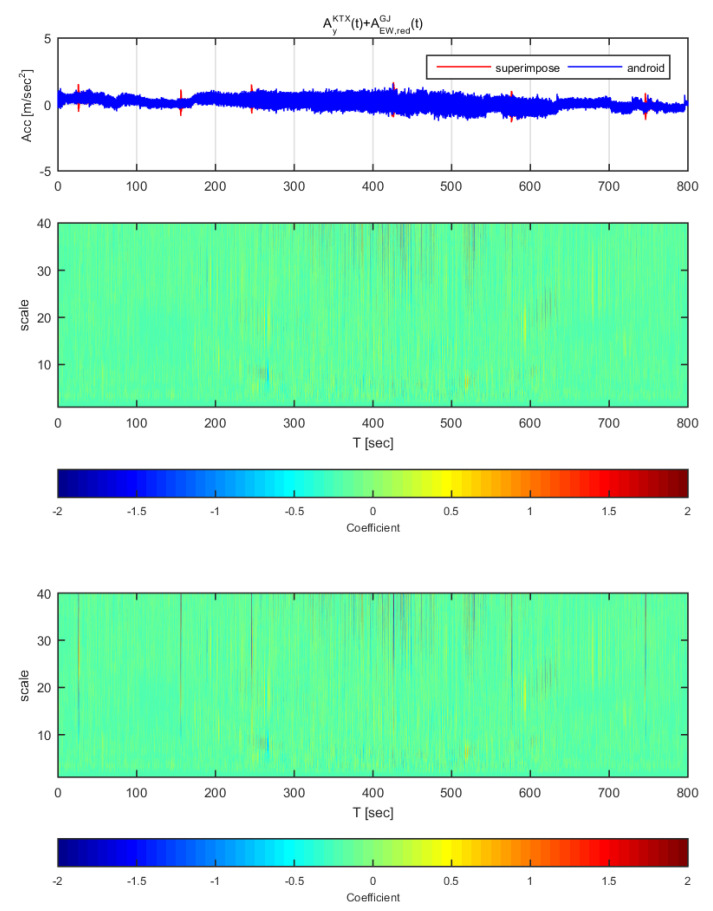
Wavelet transform coefficients for the combination of Korea Train Express (KTX) transverse direction data and Gyeongju earthquake east-west direction data (adjusted to 1 m/s^2^).

**Figure 21:**
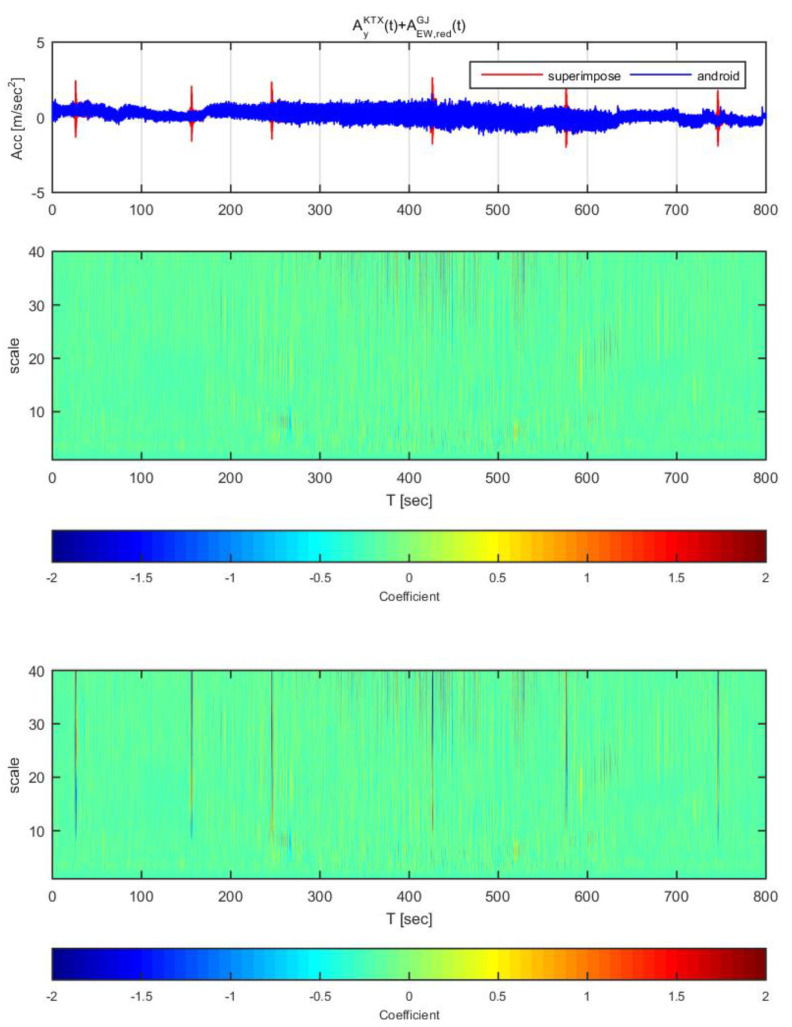
Wavelet transform coefficients for the combination of Korea Train Express (KTX) transverse direction data and Gyeongju earthquake east-west direction data (adjusted to 2 m/s^2^).

Figure 20 and Figure 21 show the time histories and the wavelet transform coefficients of the transverse direction train data and the adjusted Gyeongju earthquake data. Figure 20 displays the results for the earthquake data in the east-west direction adjusted to 1 m/s^2^ (same data as Figure 14), whereas Figure 21 shows those adjusted to 2 m/s^2^ (same data as Figure 15). In both Figure 20 and Figure 21, the train data shows significant difference when the earthquake data has been superimposed to the train data (thin lines from scale 10–40). In specific, the continuous wavelet transform results show significant difference in all sections even when the earthquake data adjusted to 1 m/s^2^. Compared to the STFT, the continuous wavelet transform detects Gyeongju earthquake signal better.

**Figure 22:**
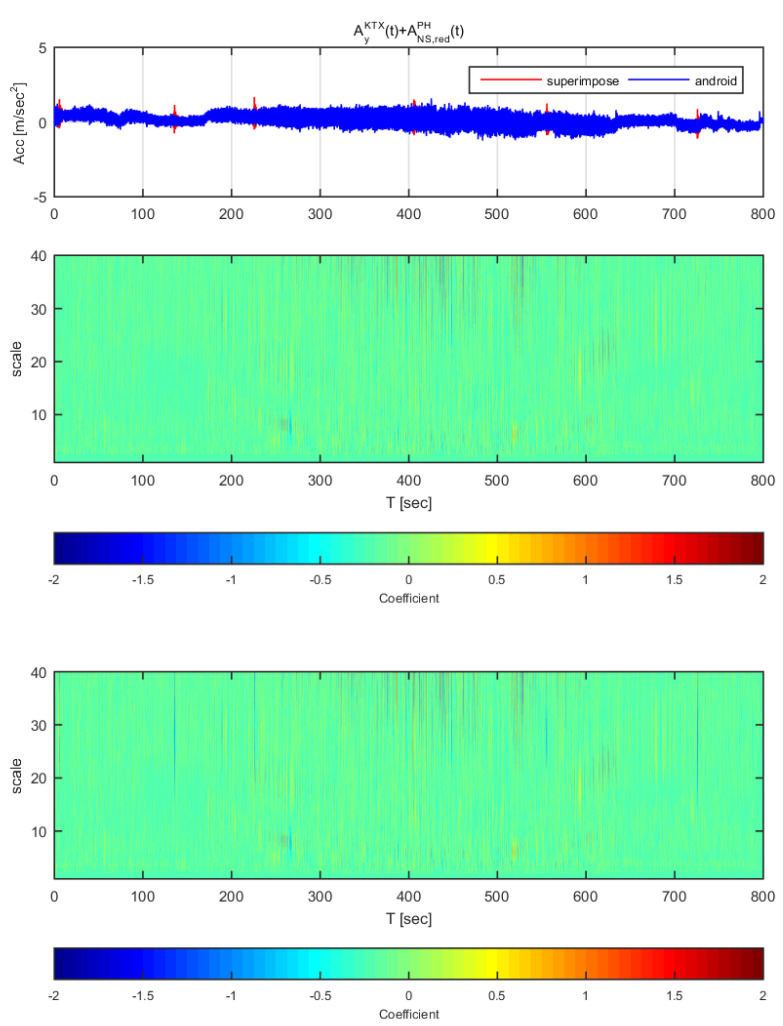
Wavelet transform coefficients for the combination of Korea Train Express (KTX) transverse direction data and Pohang earthquake north-south direction data (adjusted to 1 m/s^2^).

**Figure 23:**
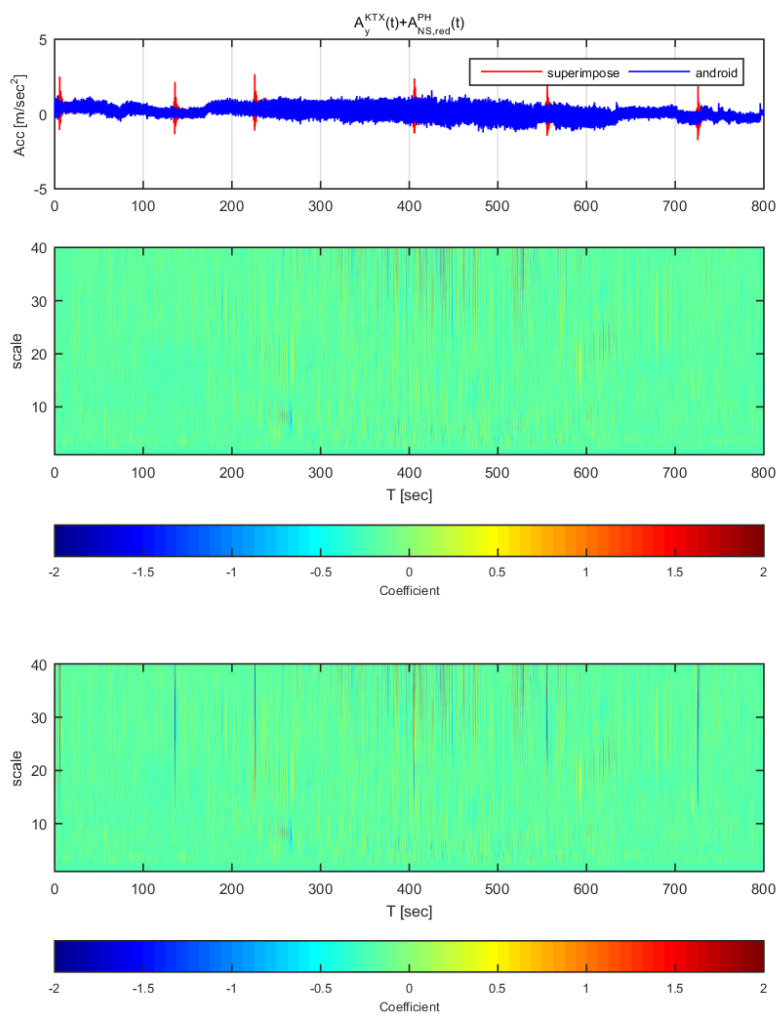
Wavelet transform coefficients for the combination of Korea Train Express (KTX) transverse direction data and Pohang earthquake north-south direction data (adjusted to 2 m/s^2^)

Figure 22 and Figure 23 show the time histories and the wavelet transform coefficients of the transverse direction train data and the adjusted Pohang earthquake data. Figure 22 displays the results for the earthquake data in the north-south direction adjusted to 1 m/s^2^ (same data as Figure 16), whereas Figure 23 shows those adjusted to 2 m/s^2^ (same data as Figure 17). In Figure 23, only the constant low speed sections exhibit a significant difference in both time history and analysis; the other sections showed no significant differences in acceleration (similar with Figure 16). While in Figure 23, all sections show significant differences in coefficients, which is similar with Figure 17. 

**Figure 24:**
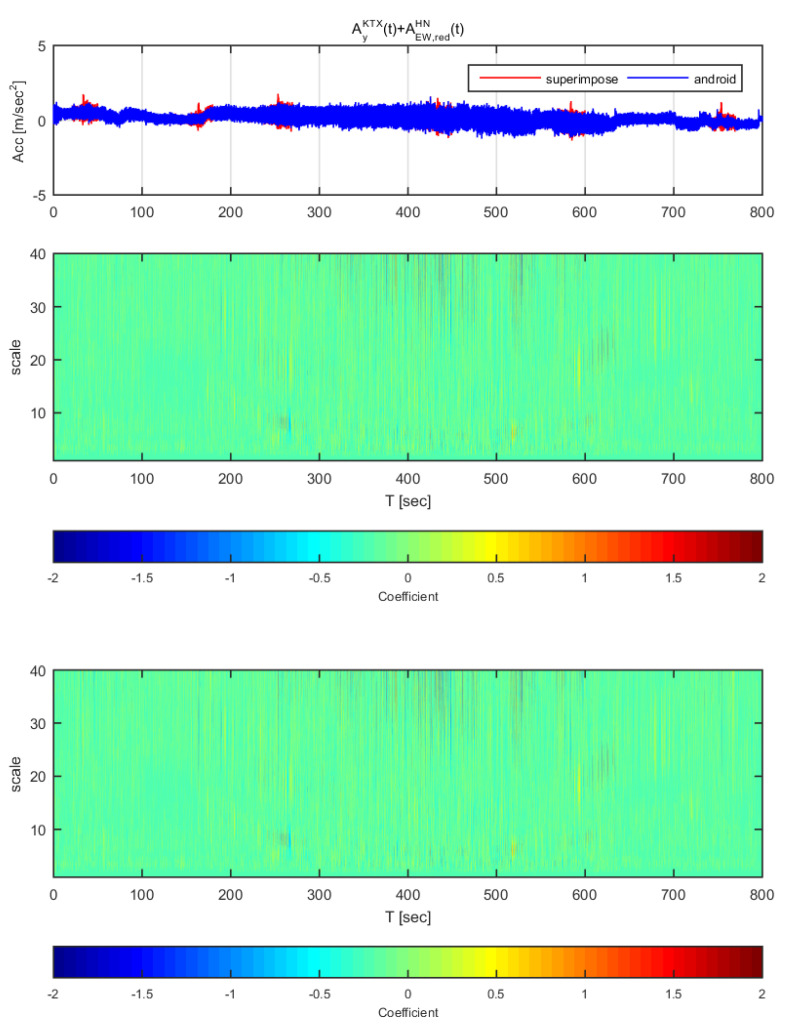
Wavelet transform coefficients for the combination of Korea Train Express (KTX) transverse direction data and Hachinohe earthquake east-west direction data (adjusted to 1 m/s^2^).

**Figure 25:**
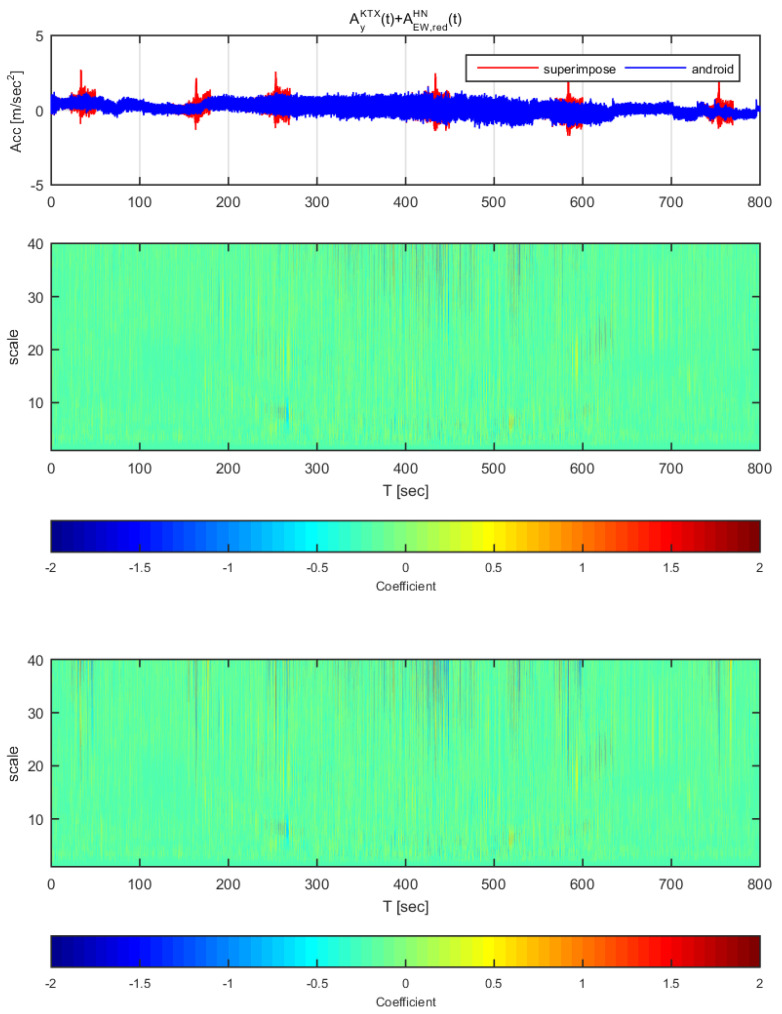
Wavelet transform coefficients for the combination of Korea Train Express (KTX) transverse direction data and Hachinohe earthquake east-west direction data (adjusted to 2 m/s^2^).

Figure 24 and Figure 25 show the time histories and the wavelet transform coefficients of the transverse direction train data and the adjusted Hachinohe earthquake data. Figure 24 displays the results for the earthquake data in the east-west direction adjusted to 1 m/s^2^ (same data as Figure 18), whereas Figure 25 shows those adjusted to 2 m/s^2^ (same data as Figure 19). In Figure 24, the difference is significantly seen in early sections with constant speed. However, other sections do not show significant differences. Similar with Figure 24, Figure 25 also shows difference in early sections. Compared to the STFT (Figure 18 and Figure 19), the continuous wavelet transform does not perform well in this case. 

## 4. Discussions

In this study, the possibility of detecting an earthquake using a smartphone on a train was evaluated in line with the pursuit of seismic detection and EEW technology development for trains. Smartphone acceleration data from a train was superimposed with scaled historical acceleration from domestic and international earthquakes to simulate an earthquake event during train operation. The data were analyzed using STFT to provide time-dependent spectral characteristics of the simulated data. The results revealed that domestic earthquakes located in relatively high frequency domains with short durations were difficult to detect intuitively by displacement curves and spectrograms, unless the energy level was above a threshold level. Although domestic earthquakes used in this study are above the threshold level, the current methodology has limits on detecting earthquakes below the threshold level with short duration. However, the Hachinohe, Japan earthquake could be detected intuitively by the smartphone due to the long duration and high energy level of the tremor, even for the scaled earthquake energy data.

The virtual earthquake detection data for the train were constructed based on the assumption that the seismic acceleration is summative with the train acceleration in the event of an earthquake. In reality, energy attenuation may occur depending on the smartphone position or type, and the frequency characteristics of the recorded train data and seismic acceleration may differ. In addition, various signals other than the acceleration of the train (e.g., walking, gaming) can be measured by an on-board smartphone. Additional to the status of smartphones, this paper simplifies the structural characteristics of the railway system as follows; first, the train is assumed to be rigidly connected to the railway. When the train is running on the railway, the train is not rigidly connected to the railway. Adding a degree of freedom between the train and the ground could provide measured data with different characteristics. Second, the railway is connected rigidly with the ground. When the train is on a bridge, the structure could amplify/attenuate signals with specific frequency range, depending of the supporting structure of the track (e.g., on the bridge with long piers). Finally, the train path is assumed to be straight and parallel or orthonormal to the seismic data. Since the railway is not straight, the virtual earthquake data would be improved if the seismic data has been modified based on the coordinate of the train path. 

Therefore, in future research, a smartphone data analysis framework will be investigated by categorizing these cases and subsequently analyzing each category. Items including the status of smartphones and users, train conditions, types and characteristics of track-supporting structures will be used to categorize cases for the investigation. For these cases, the applicability of various stochastic methods will be evaluated. Also, the effect of train-railway structural system regarding the seismic vibration transmission from the ground to the smartphone will be evaluated by the experiment in the future. In addition, a seismic detection system capable of detecting earthquakes that are difficult to manually identify will be constructed by applying machine learning tools to the STFT results to enhance this approach.
